# Association of Hyperuricemia With Immune Disorders and Intestinal Barrier Dysfunction

**DOI:** 10.3389/fphys.2020.524236

**Published:** 2020-11-27

**Authors:** Qiulan Lv, Daxing Xu, Xuezhi Zhang, Xiaomin Yang, Peng Zhao, Xuena Cui, Xiu Liu, Wan Yang, Guanpin Yang, Shichao Xing

**Affiliations:** ^1^Medical Research Center, Affiliated Hospital of Qingdao University, Qingdao, China; ^2^The Key Laboratory of Mariculture, Ministry of Education, Ocean University of China, Qingdao, China; ^3^Institute of Sports Medicine and Health, Qingdao University, Qingdao, China; ^4^Guy’s & St Thomas’ Hospital, King’s College London, London, United Kingdom

**Keywords:** hyperuricemia, gut microbiota, intestinal immune, intestinal barrier, system inflammation

## Abstract

**Background:**

More than 30–40% of uric acid is excreted via the intestine, and the dysfunction of intestinal epithelium disrupts uric acid excretion. The involvement of gut microbiota in hyperuricemia has been reported in previous studies, but the changes and mechanisms of intestinal immunity in hyperuricemia are still unknown.

**Methods:**

This study developed a urate oxidase (Uox)-knockout (Uox–/–) mouse model for hyperuricemia using CRISPR/Cas9 technology. The lipometabolism was assessed by measuring changes in biochemical indicators. Furthermore, 4-kDa fluorescein isothiocyanate–labeled dextran was used to assess gut barrier function. Also, 16S rRNA sequencing was performed to examine the changes in gut microbiota in mouse feces. RNA sequencing, Western blot, Q-PCR, ELISA, and immunohistochemical analysis were used for measuring gene transcription, the number of immune cells, and the levels of cytokines in intestinal tissues, serum, kidney, liver, pancreas, and vascellum.

**Results:**

This study showed that the abundance of inflammation-related microbiota increased in hyperuricemic mice. The microbial pattern recognition–associated Toll-like receptor pathway and inflammation-associated TNF and NF-kappa B signaling pathways were significantly enriched. The increased abundance of inflammation-related microbiota resulted in immune disorders and intestinal barrier dysfunction by upregulating TLR2/4/5 and promoting the release of IL-1β and TNF-α. The levels of epithelial tight junction proteins occludin and claudin-1 decreased. The expression of the pro-apoptotic gene *Bax* increased. The levels of LPS and TNF-α in systemic circulation increased in hyperuricemic mice. A positive correlation was observed between the increase in intestinal permeability and serum levels of uric acid.

**Conclusion:**

Hyperuricemia was characterized by dysregulated intestinal immunity, compromised intestinal barrier, and systemic inflammation. These findings might serve as a basis for future novel therapeutic interventions for hyperuricemia.

## Introduction

Hyperuricemia is a systemic disease caused by purine metabolic disorder. It is often a result of gout and renal disease, and accompanied by metabolic syndrome (Johnson et al., 2013; Debosch et al., 2014; Abeles, 2015). Lu et al. (2018) showed that hyperuricemia was associated with metabolic disorders in a urate oxidase (Uox)-knockout mouse model. Both clinical and basic studies supported the hypothesis that hyperuricemia was an independent risk factor for hypertension and cardiovascular diseases. The prevalence of hypertension, obesity, and hypertriglyceridemia in hyperuricemia is 69.1, 62.9, and 53.7%, respectively (Bardin and Richette, 2017). Hyperuricemia has become increasingly common worldwide with changes in diet and lifestyle. The prevalence of hyperuricemia has reached 21.4% in the United States and 13.3% in mainland China. Numerous anti-hyperuricemia drugs targeted at kidneys have been developed; monotherapy seriously exerts adverse effects on renal function. Hence, diverse and innovative strategies against hyperuricemia are urgently needed.

The gut is one of the largest endocrine organs, harboring thousands of microbial communities in the body. The dysbiosis of gut microbiota and immunity is associated with numerous metabolic diseases. Recent studies on the pathogenic mechanisms of hyperuricemia focus on the gut. More than 30–40% of uric acid is excreted by the intestine, and defects in intestinal clearance result in hyperuricemia (Atsushi et al., 2012; Hosomi et al., 2012). It has been established that gut microbiota participate in the metabolism of purine and uric acid (Crane, 2013; John et al., 2013). Studies have also shown that gut microbiota was considerably altered in patients with gout (Crane, 2013; Guo et al., 2016; Shao et al., 2017). These findings suggest that the gut could be a promising new therapeutic target for hyperuricemia. However, whether gut microbiota have biological effects on hyperuricemia is still unknown. Microbiota dysbiosis has a major effect on mucosal immunity, resulting in increased intestinal permeability. A weakened intestinal barrier allows the leakage of gut bacteria, inflammatory cytokines, and bacterial products such as LPS across the barrier and into the systemic circulation, which further interacts with multiple organs to cause systemic inflammation and worsen the situation (Winer et al., 2016). However, whether the changes in the microbiota in mouse models of hyperuricemia could result in alterations in intestinal immunity remains unclear. Thus, a better understanding of changes in intestinal immunity in hyperuricemia is urgently needed.

## Materials and Methods

### Animals and Models

All experiments involving mice were carried out in accordance with the protocols approved by the Animal Research Ethics Committee of the Affiliated Hospital of Qingdao University. This study established a Uox-knockout (Uox–/–) mouse model for hyperuricemia using CRISPR/Cas9 technology on C57BL/6J background. Six homozygous mice (Uox–/–) and six wild-type mice (WT) were used for experiments. The mice were housed in sterile and ventilated cages at 23°C and under a 12-h light-dark cycle. Throughout the study, the mice received a standard chow diet and water. Blood samples were collected by retro-orbital bleeding after fasting overnight. The levels of biochemical indicators, including serum uric acid, triglyceride (TC), total cholesterol (TG), and high- and low-density lipoprotein cholesterol (HDL and LDL), were determined using an automatic biochemical analyzer (Toshiba, Tokyo, Japan). Fecal samples were collected between 8 and 9 AM. The mice were weighed every week and sacrificed at an age of 15 weeks (six mice for each group). The kidney, liver, vascellum, pancreas, jejunum, ileum, and colon were sampled. Under basal conditions, mice with a weight loss of 15% under basal conditions were excluded from the study.

### 16S rRNA Gene Sequencing and Gut Microbiota Analysis

Frozen fecal samples were processed for DNA isolation, and the variable region V1–V3 of 16S rRNA was amplified using the primers 27F (5′-AGA GTT TGA TCC TGG CTC AG-3′) and 533R (5′-TTA CCG CGG CTG CTG GCA C-3′). PCR was performed by denaturing at 98°C for 30 s, followed by 25 cycles of denaturation at 98°C for 10 s, annealing at 55°C for 30 s, extension at 72°C for 30 s, and a final extension at 72°C for 5 min. The product was subsequently pooled and purified (standard gel extraction kits, Qiagen) and by Beijing Biomarker technology. Sequence data were screened and filtered for quality, and the taxonomical classification was performed using the Ribosomal Database Project (RDP)-classifier against the Greengenes reference database (Ye, 2011; Stearns et al., 2015). Sequences sharing 97% nucleotide sequence identity were clustered into operational taxonomic units (97% ID OTUs). NMDS plot for testing beta-diversity was generated in QIIME. The linear discriminant analysis effect size (LEfSe) was used for detecting discrepant bacteria among groups, and bar plots were made in R^1^. Raw sequencing data of the V1–V3 region of the 16S rRNA gene and the accompanying information were available in the Sequence Read Archive database under accession number: PRJNA600173.

### RNA Isolation and RNA Sequencing

Total RNA was isolated using TRIzol reagent (Life Technologies, United States). The mRNA was captured with Dynabeads oligo (dT) (Life Technologies). The samples were sequenced using the BGISEQ-500 platform, and about 24.08 M reads per sample were generated. Clean reads were mapped to the reference genome using HISAT^2^. The clean reads were mapped to reference transcripts using Bowtie2^3^, and then the gene expression level was calculated for each sample with RSEM^4^. KEGG pathway classification and functional enrichment were performed.

### Western Blot Analysis

Tissues were cut into small pieces and homogenized in radioimmunoprecipitation assay (RIPA) buffer containing 1% PMSF on ice. The supernatant was collected after centrifugation at 14,000 rpm for 15 min. Then, 90 μg protein was loaded onto 15 or 10% sodium dodecyl sulfate–polyacrylamide gel electrophoresis and transferred onto polyvinylidene fluoride membranes (Millipore, MA, United States). The membranes were blocked for 2 h at room temperature with 5% skimmed milk and then incubated with the following primary antibodies: mouse anti-TLR4 (Cat# sc-293072, RRID:AB_10611320, 1:500, Santa Cruz Biotechnology, United States), mouse anti-TLR5 (Cat# NB100-80842, RRID:AB_2205149 1:500, Novus, United States), rabbit anti-TLR2 (ab213676, 1:200, Abcam, United States), rabbit anti-IL-1β (Cat# ab9722, RRID:AB_308765, 1:1000, Abcam), rabbit anti-TNF-α (Cat# ab6671, RRID:AB_305641, 1:500, Abcam), mouse anti-occludin (Cat# 33-1500, RRID:AB_2533101, 1:1500, Thermo Fisher Scientific, United States), and rabbit anti-claudin-1 (Cat# NBP1-77036, RRID:AB_11026235, 1:1000, Novus) overnight at 4°C. The membranes were washed with TBST three times and incubated with anti-mouse IgG and horseradish peroxidase (HRP)-conjugated secondary antibodies (Cat# 7076, RRID:AB_330924, 1:6000, CST, United States) or anti-rabbit IgG and HRP-linked antibody (Cat# 7074, RRID:AB_2099233, 1:6000, CST, United States) at room temperature (RT) for 1 h. The protein signals were detected on an imaging system using a chemiluminescence kit (Millipore, MA, United States) and analyzed using Image-Pro Plus software (Bio-Rad, United States). Image-Pro Plus software was used to perform the gray-scale of Western Blot for the semiquantitative. Protein expression normalized to that of β-actin.

### RNA Extraction and Real-Time Quantitative PCR

Total RNAs from the kidney, liver, pancreas, vascellum, ileum, jejunum, and colon were extracted using TRIzol reagent (Life Technologies, United States) following the manufacturer’s protocols. The cDNA was synthesized using a PrimeScript RT reagent kit (Takara, Tokyo, Japan) following the manufacturer’s instructions. Real-time PCR was performed in triplicate on an ABI 7500 Fast System using SDS software with an SYBR Premix Ex Taq kit (Takara, Kyoto, Japan). The specific primers were designed using Primer 5 software and synthesized by Sangon Biotech (Table 1). All primers were verified for the production of a single specific PCR product with a melting curve program. The fold increase in mRNA abundance was calculated by the 2^–ΔΔCt^ method and normalized to GAPDH as an internal control.

**TABLE 1:** The sequences of the primers.

Names	Primers	Sequence	Species
NOD1	Forward (5′→3′)	CGGCAGCGGAAGTGGAAGAAG	Mice
	Reverse (5′→3′)	GTGTTGACTCAGTCTCGCTTCCTC	
NOD2	Forward (5′→3′)	GTGTTGACTCAGTCTCGCTTCCTC	Mice
	Reverse (5′→3′)	GTGTCGGCATCTCTGTTCAGGTG	
TLR4	Forward (5′→3′)	GCAGAAAATGCCAGGATGATG	Mice
	Reverse (5′→3′)	AACTACCTCTATGCAGGGATTCAAG	
TLR2	Forward (5′→3′)	CTCCCACTTCAGGCTCTTTG	Mice
	Reverse (5′→3′)	AGGAACTGGGTGGAGAACCT	
TLR3	Forward (5′→3′)	CAGGCGTCCTTGGACTTGAAGC	Mice
	Reverse (5′→3′)	TGCTGAACTGCGTGATGTACCTTG	
TLR5	Forward (5′→3′)	CCACCGAAGACTGCGATGAAGAG	Mice
	Reverse (5′→3′)	CCAGACCTTGTCCTTGAACACCAG	
TLR7	Forward (5′→3′)	ATCGTGGACTGCACAGACAAGC	Mice
	Reverse (5′→3′)	AGCCTACGGAAGGAATCTGGAGAG	
TLR9	Forward (5′→3′)	GACTTCAGCGGCAACGGTATGG	Mice
	Reverse (5′→3′)	TAGTTGTCTCGGAGGCTCAGCAG	
NLRP3	Forward (5′→3′)	ATGCTGCTTCGACATCTCCT	Mice
	Reverse (5′→3′)	AACCAATGCGAGATCCTGAC	
Occludin	Forward (5′→3′)	TTGAAAGTCCACCTCCTTACAGA	Mice
	Reverse (5′→3′)	CCGGATAAAAAGAGTACGCTGG	
ZO-1	Forward (5′→3′)	ACTCCCACTTCCCCAAAAAC	Mice
	Reverse (5′→3′)	CCACAGCTGAAGGACTCACA	
Bak1	Forward (5′→3′)	GGTCTTTCGAAGCTACGTTTTT	Mice
	Reverse (5′→3′)	ATCTTGGTGAAGAGTTCGTAGG	
Bax	Forward (5′→3′)	CCATGATGGTTCTGATCAGCTC	Mice
	Reverse (5′→3′)	TTGCCCTCTTCTACTTTGCTAG	
Bcl2l10	Forward (5′→3′)	TGACTACATATTCTTCTGCGC	Mice
	Reverse (5′→3′)	CTTTGGAGAGCAACTTATCTGC	
GAPDH	Forward (5′→3′)	AAATGGTGAAGGTCGGTGTGAACG	Mice
	Reverse (5′→3′)	ATCTCCACTTTGCCACTGC	
	Reverse (5′→3′)	ACA TTG GGG GTA GGA ACA CGG A	

### Hematoxylin-Eosin and Immunohistochemical Analyses

Histopathological analysis was carried out using hematoxylin-eosin (H&E) on samples from the ileum, jejunum, and colon of WT and Uox^–/–^ mice. The tissues were formalin-inflated and then paraffin-embedded. The paraffin-embedded tissue blocks were stained with H&E, and epithelial architecture was scored and mounted on an optical microscope (Olympus, Japan).

For immunohistochemical analysis, the tissue was blocked with 5% goat serum, containing 0.25% Triton-100 for 1 h at RT and incubated with primary antibodies against CD68 (1:200, Santa Cruz Biotechnology), CD3 (1:200, Abcam), and claudin-1 (1:200, Santa Cruz Biotechnology) at 4°C overnight. The tissue that was not incubated with specific primary antibodies was used as a negative control. The slices were incubated with specific secondary antibodies (1:1000, CST) for 1 h at RT after washing with PBS three times. Images were then obtained with an Olympus Provis AX70 microscope (Olympus, Japan).

### Measuring the Permeability of the Epithelial Barrier Using FITC-Labeled Dextran

The 4-kDa fluorescein isothiocyanate (FITC)-labeled dextran (FD4; Sigma–Aldrich) was used to assess *in vivo* intestinal permeability. The mice were deprived of water for 4 h prior to an oral gavage (40 mg/100 g body weight) with FITC-labeled dextran at a concentration of 80 mg/mL in PBS. After 4 h, blood was collected by retro-orbital bleeding and centrifuged at 2000 rpm for 10 min. The serum was collected, and fluorescence intensity was measured on fluorescence plates using an excitation wavelength of 485 nm and an emission wavelength of 528 nm.

### Measurement of the Serum Levels of LPS, IL-1β, and TNF-α

Serum levels of LPS, IL-1β, and TNF-α were measured using an ELISA kit (Mibio, China) in duplicate (*n* = 6) following the manufacturer’s instructions. Briefly, plates with specific antibodies were incubated with serum or tissue homogenates, washed, and incubated with HRP-avidin before quantification. The absorbance at 405 nm was measured with a microplate reader. The total proteins in the kidney, liver, pancreas, and vascellum were detected using a BCA Protein Assay kit (Thermo, United States). The contents of IL-1β and TNF-α in tissues were normalized to total protein.

### Statistical Analysis

All data were displayed as the mean ± standard error of the mean (SEM). Hyperuricemia and control groups were compared using the Student unpaired-sample *t*-test, and comparisons of more than three groups were analyzed by analysis of variance with Tukey’s *post hoc* test using SPSS 17.0. *P*-values < 0.05 indicated statistically significant differences.

## Results

### Lipometabolism Was Disordered in Hyperuricemic Mice

**FIGURE 1:**
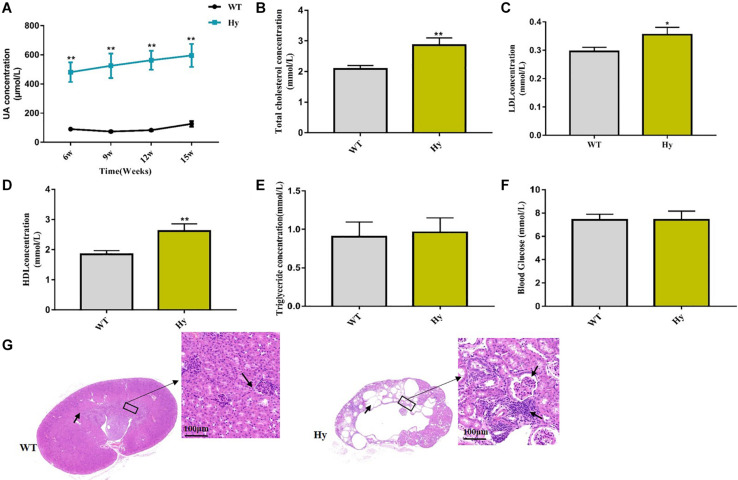
Lipometabolism was disordered in hyperuricemic mice. **(A–F)** Mice fasted overnight, and blood samples were collected at the age of 15 weeks. Serum uric acid **(A)**, TC **(B)**, LDL **(C)**, HDL **(D)**, TG **(E)**, and glucose **(F)** levels were detected using an automatic biochemical analyzer. **(G)** HE staining analysis of the architecture of kidneys with normal tubules and glomeruli and abnormal kidneys with collapsed and necrotic nephrons marked with an arrow (magnification, 200×, scale bar = 100 μm). WT, wild-type mice; Hy, hyperuricemic mice (*n* = 6 mice per group). Mean ± SEM were plotted. Statistical significance was determined using the Student unpaired-sample *t*-test (*P < 0.05 and **P < 0.001).

This study used a mouse model featuring Uox genetic dysfunction, leading to hyperuricemia. Significant differences were detected in the serum levels of uric acid between the two groups (Figure 1A). The serum levels of TC and LDL increased in hyperuricemic mice, suggesting a risk of hyperuricemia for patients with cardiovascular disease and stroke (Figures 1B,C). An increase in the HDL level was observed (Figure 1D), indicating disorders of lipid metabolism. However, no changes in triglyceride and blood glucose levels were found between hyperuricemic and WT mice (Figures 1E,F). The morphology of the kidney in hyperuricemic mice was significantly abnormal, including collapsed and necrotic nephrons, interstitial edema, and inflammatory cell infiltration (blue dot-shaped cells marked with an arrow). We also noted lamellar necrosis of epithelial cells in renal tubules (Figure 1G).

### Microbiota Composition Was Altered in Hyperuricemic Mice

**FIGURE 2:**
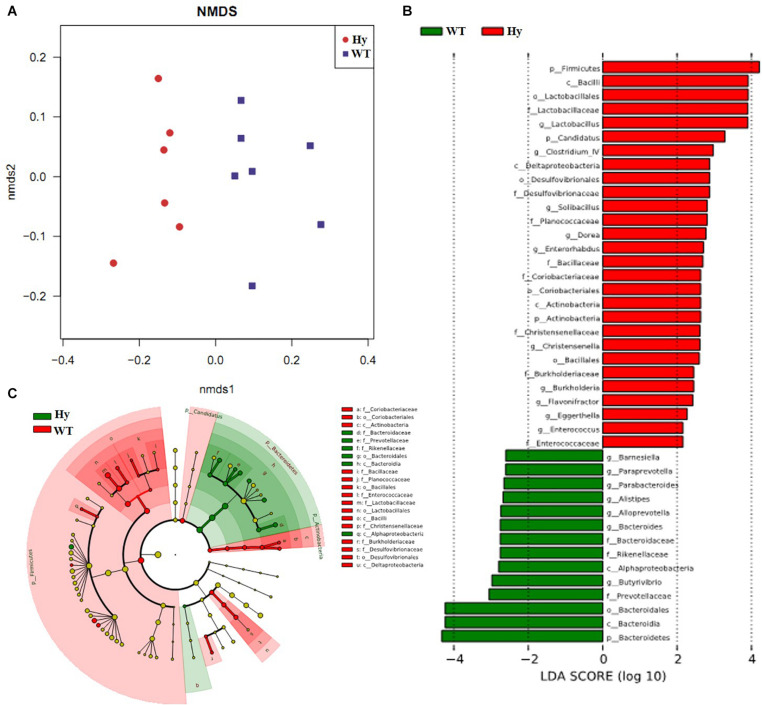
Microbial dysbiosis occurred in hyperuricemic mice. The taxa summary of microbiota was achieved using the Ribosomal Database Project (RDP)-classifier. Sequences sharing 97% nucleotide sequence identity were clustered into operational taxonomic units (97% ID OTUs). **(A)** The community composition was analyzed using NMDS based on unweighted UniFrac distance; **(B,C)** The microbiota composition was analyzed using LEfSe (*n* = 7). WT, wild-type mice; Hy, hyperuricemic mice.

Preclinical animal models of hyperuricemic mice featuring Uox genetic dysfunction were used to investigate the causative link between altered microbiota and gut pathology. As showed by the results of this study, Hyperuricemic mice had microbiota dysbiosis. NMDS based on unweighted UniFrac distance showed two separate clusters with highly distinct community composition (Figure 2A). The discrepant bacterial taxa between the two groups were further analyzed using LEfSe analysis. As shown in Figures 2B,C, the abundance of *Bacteroides*, *Alloprevotella*, and intestinal inflammation–associated bacteria, such as *Alistipes* and *Parabacteroides*, significantly increased in hyperuricemic mice, while the major butyrate-producing genera, such as *Clostridium*, and protective bacterium *Lactobacillus*, and anaerobic bacteria such as *Candidatus* and members of Coriobacteriaceae, were depleted.

Finally, the correlation between the uric acid level and bacterial abundance was analyzed using Pearson correlation coefficients with *P* < 0.05 to preliminarily verify the association between microbiota and hyperuricemia. Five (*Alloprevotella, Alistipes, Butyrivibrio, Bacteroides*, and *Barnesiella*) and three (*Clostridium_XlVa, Burkholderia*, and *Christensenella*) bacterial genera having a positive and negative correlation with uric acid, respectively, were identified in the present study (Table 2). The bacteria having a positive correlation with uric acid were also associated with intestinal inflammation. These results demonstrated that the altered microbiota was a signature of gut inflammation.

**TABLE 2:** The correlation between bacteria and UA content in mouse.

Gut bacteria	Mouse	
	*R* value	Significance level
*Christensenellaceae*	−0.62	*
*Coriobacteriaceae*	−0.7	**
*Bacteroidaceae*	0.6	*
*Rikenellaceae*	0.68	*
*Prevotellaceae*	0.62	*
*Planococcaceae*	−0.78	**
*Desulfovibrionaceae*	−0.72	**
*Lactobacillaceae*	−0.74	**
*Streptococcaceae*	−0.64	*
*Burkholderiaceae*	−0.61	*
*Enterococcaceae*	−0.61	*
*Bacteroides*	0.6	*
*Alloprevotella*	0.74	**
*Alistipes*	0.74	**
*Butyrivibrio*	0.64	*
*Barnesiella*	0.56	*
*Clostridium_XlVa*	−0.67	*
*Burkholderia*	−0.61	*
*Christensenella*	−0.62	*

Asterisks, the significance of discrepancy, **P < 0.01, *P < 0.05.

### Gut Microbiota Disturbed Intestinal Immunity

**FIGURE 3:**
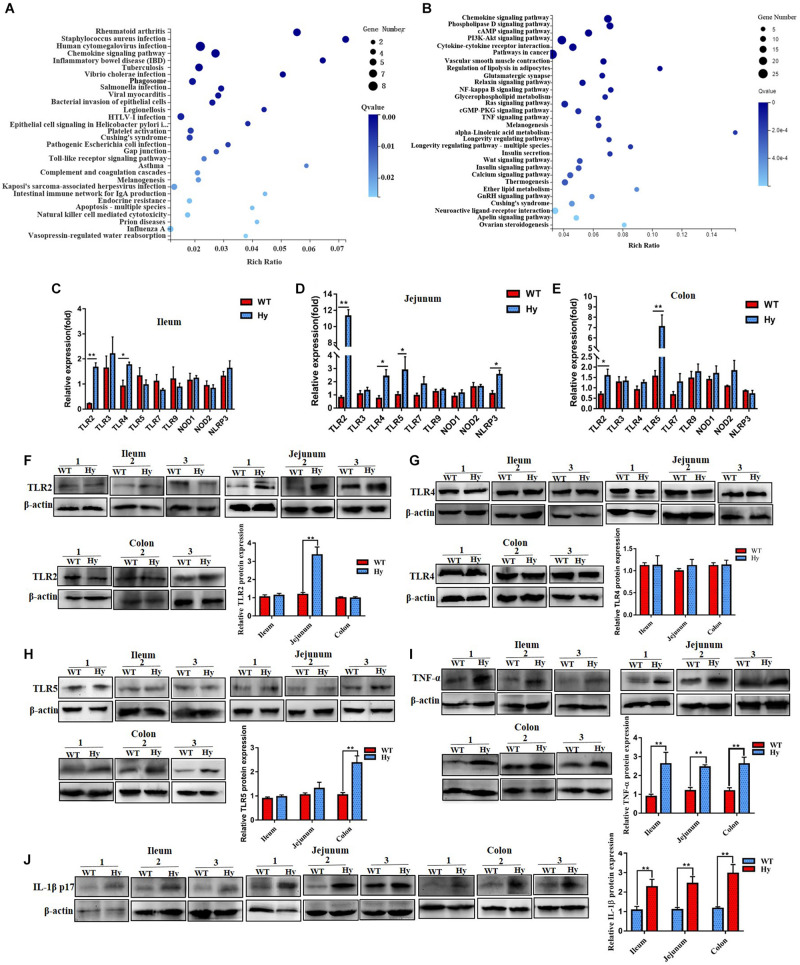
Hyperuricemic mice had increased intestinal inflammation. **(A,B)** The most pathway functional enrichment of DEGs is associated with bacterial infectious diseases and signal transduction pathways. The *x*-axis represents the enrichment factor. The *y*-axis represents the pathway name. The color indicates the *q*-value (high: white; low: blue), and the lower *q*-value indicates more significant enrichment. Point size indicates the DEG number (the bigger dots refer to a larger amount). Rich factor refers to the value of enrichment factor, which is the quotient of the foreground value (the number of DEGs) and the background value (total gene amount). The larger the value, the more significant the enrichment. **(C–E)** Gene expression of TLRs and NLRs in the ileum **(B,C)**, jejunum **(D)**, and colon **(E)** was determined using Q-PCR (*n* = 6 mice per group). **(F–H)** Protein density of TLR2 **(F)**, TLR4 **(G)**, and TLR5 **(H)** from the ileum, jejunum, and colon tissue was determined using Western blot analysis. **(I,J)** Expression of TNF-α **(I)** and IL-1β **(J)** in the ileum, jejunum, and colon was verified by Western blot analysis. Data represent three independent experiments. Mean ± SEM were plotted. Statistical significance was determined using the Student unpaired-sample *t*-test (*P < 0.05 and **P < 0.001).

The abundance of inflammation-associated bacteria increased in hyperuricemic mice, especially in the intestine. RNA sequencing was performed to compare intestinal mRNA levels between WT and hyperuricemic mice to investigate the mechanisms by which gut microbiota affected intestinal epithelial function. Pathway functional enrichment was explored through KEGG pathway enrichment to highlight the DEGs relevant to bacterial infectious diseases between WT and hyperuricemic mice. As shown in Figures 3A,B, microbial pattern recognition associated with the Toll-like receptor pathway and inflammation-associated TNF and NF-kappa B signaling pathways were enriched. Furthermore, several DEGs associated with bacterial infectious diseases, such as *Staphylococcus aureus, Salmonella*, and pathogenic *Escherichia coli*, were also enriched. Moreover, inflammatory bowel disease– and bacterial invasion-associated DEGs were also significantly enriched. The findings were confirmed using Q-PCR. We evaluated changes in the levels of microbial pattern recognition receptors in the ileum, jejunum, and colonic tissue. As shown in Figures 3C–E, the genes whose expression most strongly upregulated in hyperuricemic mice were TLR2, TLR4, and TLR5, which are indicative of gut dysbiosis. The expression of TLR2, TLR4, TLR5, and NLRP3 was significantly aberrant, especially in the jejunum. However, the protein level of TLR4 remained unchanged in the ileum, jejunum, and colon. The level of only TLR2 protein increased in the jejunum, and the level of TLR5 protein increased in the colon (Figures 3F–H). The levels of pro-inflammatory cytokines were further determined using Western blot analysis. As shown in Figures 3I,J, the augmented expression of TNF-α was observed in the ileum, jejunum, and colon. Furthermore, the level of inflammatory bowel disease-associated factor IL-1β was also elevated in the ileum, jejunum, and colon. These data indicated that the altered microbiota composition in hyperuricemia was accompanied by changes in intestinal immunity.

### Hyperuricemia Was Characterized by Compromised Intestinal Barrier and Infiltration of Lymphocytic Cells

**FIGURE 4:**
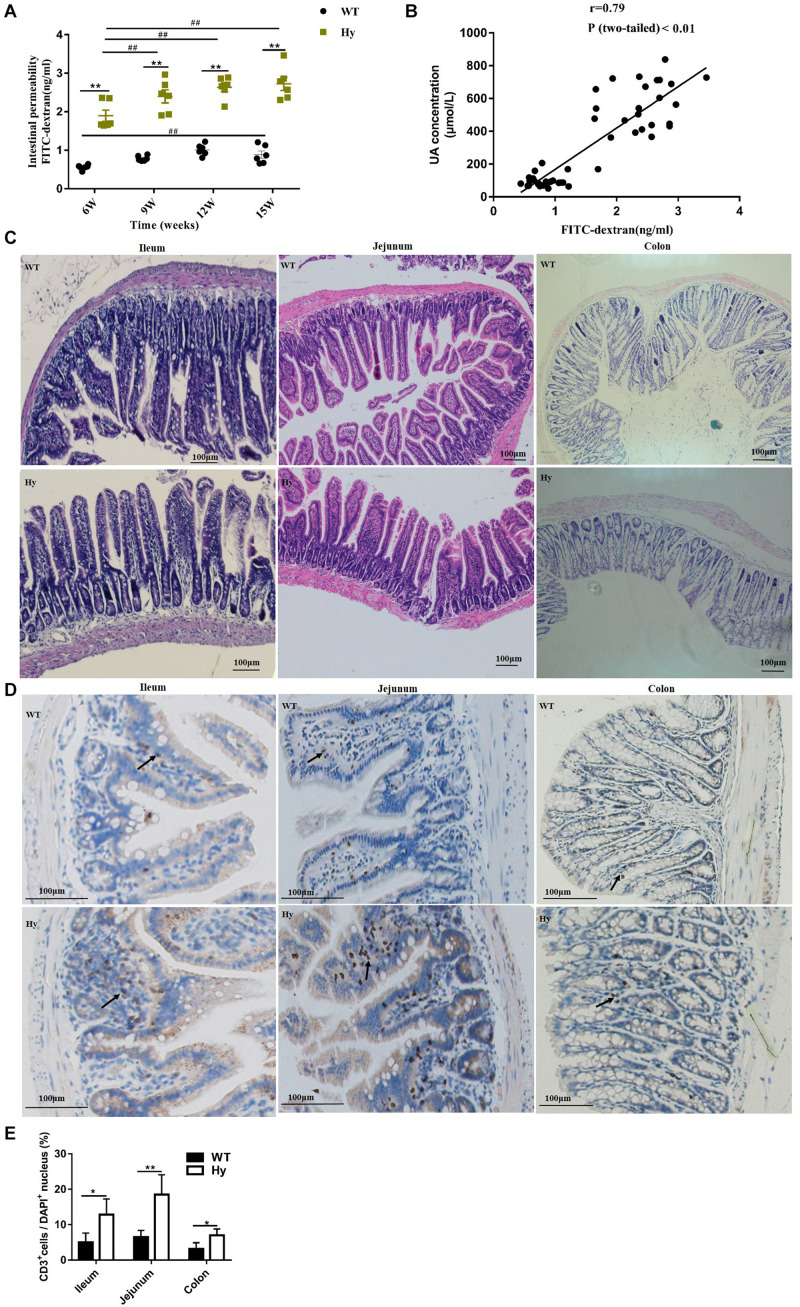
Hyperuricemia was associated with intestinal barrier dysfunction. **(A)** Intestinal permeability was measured in WT and Hy mice at the age of 6, 9, 12, and 15 weeks by oral gavage with FD4, and fluorescence translocation into the plasma were determined. **(B)** Correlation of intestinal permeability with the uric acid level was analyzed using Pearson correlation coefficients with *P* < 0.05. **(C)** Epithelial architecture of the ileum, jejunum, and colon, analyzed by staining with H&E (100× magnification). **(D)** Representative image of immunohistochemical staining for CD3+ (brown) in the ileum, jejunum, and colon. Arrows indicate positive cells. All photos were taken at 100×, scare bar = 100 μm. **(E)** Quantification data of CD3^+^ cells. The number of CD3^+^ cells was counted manually using the Image J 1.47v software, and the percentage of CD3^+^ cells per DAPI^+^ cells was calculated (*n* = 6 mice per group). Mean ± SEM were plotted. Statistical significance was determined using ANOVA with Tukey’s *post hoc* test. *P < 0.05 and **P < 0.001 versus WT; ^#^*P* < 0.05 and ^##^*P* < 0.001 versus 6 weeks.

The association of intestinal inflammation and epithelial barrier dysfunction with hyperuricemia was further examined. The concentration of serum FD4 was detected after 6, 9, 12, and 15 weeks. As shown in Figure 4A, a feature common to hyperuricemic mice at different time points was a compromised barrier. Notably, older mice had higher intestinal permeability in both hyperuricemia and WT groups. The intestinal permeability increased with age in both hyperuricemia and WT mice. However, the increase was robust in the hyperuricemia group, but slight in the WT group. It seemed that age might predispose hyperuricemic mice to the exacerbation of intestinal barrier dysfunction. A positive correlation was observed between increased intestinal permeability and uric acid levels (*r* = 0.79, *P* < 0.01), indicating that increased intestinal permeability might contribute to hyperuricemia (Figure 4B). Together, these observations indicated that hyperuricemia was characterized by a compromised intestinal barrier. The epithelial structure was stained with H&E to further examine pathological changes in the intestinal mucosa. However, no gross differences were found in the intestinal architecture of the ileum, jejunum, and colon (Figure 4C). This study further examined inflammatory cell infiltration in the intestine. As shown in Figures 4D,E, significant lymphocytic (CD3^+^) infiltration was found in the ileum, jejunum, and colon in the hyperuricemic mice compared with the WT mice, revealing the infiltration of lymphocytic cells into the intestinal epithelium.

### Molecular Mechanisms That Involved in a Compromised Intestinal Barrier

**FIGURE 5:**
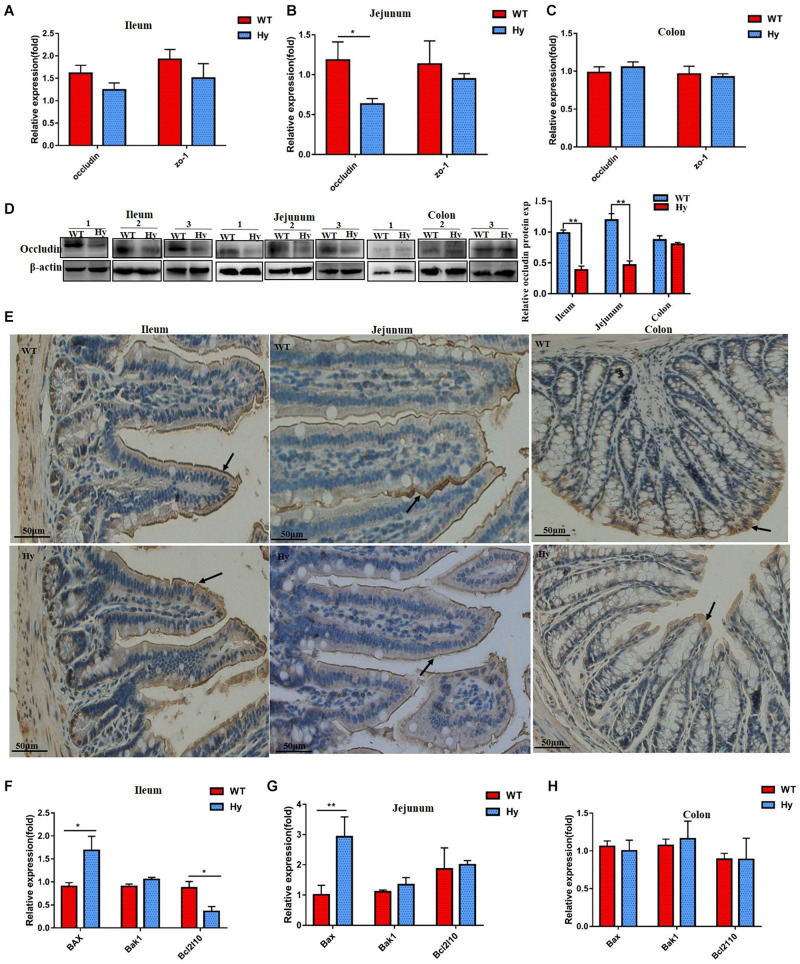
Hyperuricemia altered the expression of tight junction proteins. **(A–C)** Gene expression of occludin and ZO-1 in the ileum **(A,B)**, and colon **(C)** was determined using Q-PCR. **(D)** Protein density of occludin from the ileum, jejunum, and colon tissues was determined using Western blot analysis. Data represent three independent experiments. **(E)** Immunohistochemical staining for claudin-1 (brown, marked with arrows) in the ileum, jejunum, and colon. All photos were taken at 200×, scare bar = 50 μm. **(F–H)** Expression of anti-apoptotic protein BCL-B (Bcl2l10) and pro-apoptotic genes Bak1 and Bax in the ileum **(F)**, jejunum **(G)**, and colon **(H)** was determined using Q-PCR (*n* = 6 mice per group). Mean ± SEM were plotted. Statistical significance was determined using the Student unpaired-sample *t*-test (*P < 0.05 and **P < 0.001).

The expression of epithelial tight junction proteins was assessed to further investigate the molecular mechanisms involved in a compromised intestinal barrier. We measured the mRNA expression of occludin and ZO-1. As shown in Figures 5A–C, the expression of the occludin gene was lower only in the jejunum, while ZO-1 mRNA levels were unaffected. However, the protein expression of occludin was markedly attenuated in the ileum and jejunum but slightly changed in the colon. The expression of tight junction proteins occludin and claudin-1 were also lower in the jejunum and colon (Figures 5D,E). The present study further analyzed the expression of pro- and anti-apoptotic genes. As shown in Figures 5F–H, the expression of the pro-apoptotic gene *Bax* significantly increased in both the ileum and jejunum, while the expression of the anti-apoptotic gene *Bcl2*l reduced in the ileum. The expression of *Bak1* remained unchanged.

### Compromised Barrier Was Associated With Increased Systemic Inflammation

**FIGURE 6:**
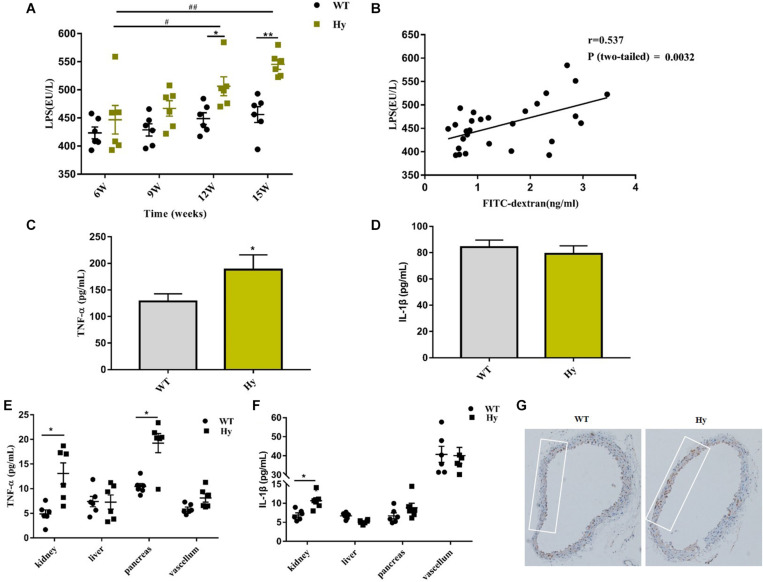
Hyperuricemia drove the translocation of LPS and increased circulating inflammation. **(A)** Serum LPS levels in WT and Hy mice (6, 9, 12, and 15 weeks old) were detected using ELISA in duplicate. **(B)** Correlation of intestinal permeability and LPS was analyzed using Pearson correlation coefficients. **(C,D)** TNF-α **(C)** and IL-1β **(D)** levels in the serum of WT and Hy mice were detected using ELISA. **(E,F)** Mice were sacrificed at the age of 15 weeks. The kidneys, liver, pancreas, and vascular tissue were homogenized, and TNF-α **(E)** and IL-1β **(F)** levels were measured using ELISA. Please note the break in the *y*-axis. **(G)** Immunohistochemical staining for CD68^+^ (brown) in the vascular tissue (100× magnification; *n* = 6 mice per group). Mean ± SEM were plotted. Statistical significance was determined using ANOVA with Tukey’s *post hoc* test. *P < 0.05 and **P < 0.001 versus WT; ^#^*P* < 0.05 and ^##^*P* < 0.001 versus 6 weeks.

This study further explored whether the compromised barrier could result in the influx of microbial products in the system circulation. Consistently, the circulating levels of LPS were higher in hyperuricemia mice than in WT mice. Notably, the level of LPS in hyperuricemia also increased with age (Figure 6A), which was in tandem with increased intestinal permeability. However, the level of LPS did not increase at different time points in WT mice compared with hyperuricemia mice. A close positive correlation was observed between intestinal permeability and the circulating LPS level (Figure 6B). These findings indicated that a compromised barrier drove the LPS influx in the system circulation.

LPS could trigger a series of pro-inflammatory responses. Therefore, it was hypothesized that high systemic levels of LPS might provoke an overt systemic inflammation. As shown in Figure 6C, the circulating levels of TNF-α increased in hyperuricemic mice. However, the serum IL-1β levels did not increase despite high levels of LPS (Figure 6D). It was further hypothesized that the increased systemic dissemination of microbial products and pathogenic factors as a result of the impaired intestinal barrier, might have a direct effect on multiple organs. The levels of pro-inflammatory cytokines TNF-α and IL-1β were detected in various organs. As shown in Figures 6E,F, the concentration of TNF-α was significantly elevated in the kidneys and pancreas, but no change was observed in the liver and vascellum. However, the expression of IL-1β was upregulated only in the kidneys (Figures 6E,F). Furthermore, significant infiltration areas of macrophages (CD68+) were found in the vascellum (Figure 6G). These data displayed a low-grade inflammation in the kidneys, pancreas, and vascellum.

## Discussion

Accumulating evidence has emphasized the involvement of gut microbiota in hyperuricemia. However, whether the changes in microbiota result in gut pathology is still unknown. Besides, the mechanistic basis for intestinal immunity in hyperuricemia remains poorly understood. This study was novel in examining changes and the mechanism of intestinal immunity in hyperuricemic mice. Hyperuricemic mice suffered from disturbance of the intestinal flora, with an increased abundance of inflammation-associated microbiota. Hyperuricemic mice developed a pro-inflammatory pattern of intestinal immune dysregulation characterized by increased expression of TLR2/4/5, IL-1β, and TNF-α. Consequently, dysregulated intestinal immunity and gut dysbiosis coincided to disrupt the intestinal barrier and promote the influx of microbial influx in the system circulation. The study was novel in yielding a report substantiating the changes in intestinal immunity in hyperuricemic mice and suggesting target treatments from a new perspective.

The intestinal tract is home to thousands of microbial communities in the body. Changes in microbiota may cause diseases. Many studies have linked gout to gut microbial dysbiosis. In patients with gout, microbiota with the xanthine dehydrogenase gene are enriched whereas microbiota with the allantoinase gene, which degraded uric acid to urea, are depleted (Guo et al., 2016). Thus, gut microbiota might be a novel treatment target for gout. Consistent with the available data, microbiota dysbiosis occurred in hyperuricemic mice. The abundance of health-promoting probiotics or microbiota with the functional capacity of producing butyrate (such as *Clostridium* and *Lactobacillus*) (Koji et al., 2014) decreased in hyperuricemic mice in the present study. However, the prevalence of microbiota exacerbated or implicated in intestinal inflammation, such as *Alistipes* and *Parabacteroides*, increased (Bassett et al., 2015; Walujkar et al., 2018). Furthermore, the microbiota positively correlated with uric acid and was also highly correlated with intestinal inflammation. Evidence shows that microbiota affected intestinal immunity by changing the levels of anti-inflammatory species, decreasing bacterial richness, changing microbe–microbe interactions, or producing bacterial metabolites (Anne et al., 2012; Emmanuelle et al., 2013). However, previous reports highlighted only the importance of both the composition and function of microbiota but the effects of these changes on intestinal immunity remain unclear.

This study examined intestinal immune dysregulation in hyperuricemic mice, demonstrating the abnormal expression of TLR, infiltration of lymphocytes, and increased levels of pro-inflammatory cytokines in different parts of the bowel. Disturbance in the intestinal flora and increased inflammation in the intestine have also been reported in other mouse models of obesity and diabetes (Brown et al., 2013). Increased levels of pro-inflammatory cytokines, such as TNF-α, IL-1β, IL-6, and IL-12, were observed in the intestinal tissue of mouse models with obesity (Maynard et al., 2012), which was consistent with the present findings. TNF-α levels increased in both the intestinal tissue and serum of hyperuricemic mice. Winer et al. also demonstrated the important role of TNF-α in both HFD- and inflammatory bowel disease-induced barrier dysfunction. Gut immune cells have been shown with the ability to traffic lymphoid organs, such as the spleen (Morton et al., 2014). Thus, the elevated levels of circulating TNF-α in hyperuricemic might may also be derived from the bowel lumen due to the compromised intestinal barrier. TNF knockout mice showed protection against age-associated systemic inflammation (Thevaranjan et al., 2017). However, whether an equivalent effect existed in hyperuricemia as in age-associated systemic inflammation was unclear. Furthermore, among several TLRs, the TLR2 mRNA level significantly increased in every part of the intestine, especially in the jejunum. Both protein and mRNA levels increased in the jejunum. TLR2 interacted with ligands, including bacterial lipopeptides, lipoteichoic acid, and yeast zymosan, to increase apical tightening through the activation of PKC-α and PKC-δ signaling pathways. However, under pathologic conditions, it promoted intestinal inflammation (Cario et al., 2004; Schultz et al., 2007). The overexpression of TLR2 in the jejunum might predict an aggravating intestinal inflammation, which needs further investigation.

The intestinal barrier provides a physical barrier against the excessive entry of luminal microbiota or damaging agents into the systemic circulation (Farhadi et al., 2010). The balanced interaction between microbiota and intestinal immunity is important to maintain a healthy mucosal barrier function. Once the intestinal epithelial barrier is breached, multiple organs are exposed to pathogenic microbiota and products. Evidence shows intestinal barrier dysfunction is a distinctive feature of metabolic diseases (Ekihiro and Bernd, 2012; Rera et al., 2012; Kristoff et al., 2014). Hyperglycemia drives intestinal barrier permeability in mouse models of obesity and diabetes, leading to the systemic influx of microbial products and enhanced systemic infection and inflammation (Thaiss et al., 2018). Alcohol-induced tissue damage and organ dysfunction were also accompanied by gut leakiness (Patel et al., 2015). Hyperuricemia is consistently characterized by increased intestinal permeability. Notably, intestinal permeability increased with age and levels of circulating LPS in hyperuricemic mice. Clinically, elderly individuals have a high risk of suffering from hyperuricemia. However, the related mechanism remains unclear. Thevaranjan reported that intestinal permeability increased with age, leading to the entry of microbial products into the bloodstream and triggering systemic inflammation (Thevaranjan et al., 2018). These data were consistent with the findings of the present study. The study also reported a positive correlation of levels of uric acid with intestinal permeability. Increased inflammation in the intestine led to the dysfunction of the intestinal barrier and disturbed crosstalk between the host and microbiota, further affecting the transport of uric acid and aggravating the progression of hyperuricemia. Thus, increased intestinal permeability with age might be the pivotal risk factor for hyperuricemia.

Recently, growing evidence has indicated that a compromised intestinal barrier preceded systemic inflammation (Shmagel et al., 2016; Andersen et al., 2017; Rizzetto et al., 2018). Similarly, increased levels of circulating LPS and TNF-α were observed in hyperuricemic mice, indicating systemic inflammation. Multiple organs also had a state of low-grade inflammation. A marked inflammatory response was noted especially in the pancreas and vascellum. Both basic and clinical studies have evidenced hyperuricemia as an independent risk factor for insulin resistance and atherosis (Kim et al., 2018; Sunagawa et al., 2018), but they examined the mechanism only in terms of direct inflammation and increase in the uric acid level. It appeared as if the elevated serum LPS was the consequence of increased intestinal permeability in patients with insulin resistance and vascular injury. LPS is the activator of pro-inflammatory cytokines, such as TNF-α, IL-1β, and IL-6, contributing to the development of insulin resistance (Olefsky and Glass, 2010). Estes et al. (2010) demonstrated that reducing the levels of circulating LPS attenuated immune dysfunction and systemic inflammation. The present study only evaluated the level of LPS in the serum, however, it is possible that LPS also entered the organs through systemic circulation in the form of chylomicrons (Yuehui et al., 2009). No changes were found in glucose levels. It was presumed that LPS was a risk factor for diabetes, but not a directly influencing factor. The levels of HDL and LDL increased in hyperuricemic mice, which contradicted previous findings. HDL has been considered to be a so-called “good cholesterol” because it reduces the risk of clogged arteries and atherosclerosis. However, a recent study showed that extremely high levels of HDL might be associated with an increased risk of heart attack and death (Madsen et al., 2017). The present study obtained a similar result, but the mechanism was unknown. This finding might be ascribed to hyperuricemia-induced dyslipidemia. However, further studies are needed to elucidate the mechanism.

Collectively, this study was novel in clarifying the change and mechanism of intestinal immunity in hyperuricemia and demonstrating the strong correlation of hyperuricemia with gut barrier dysfunction. Furthermore, the dysfunction of the intestinal barrier and imbalance in host–microbiome crosstalk was vital in aggravating hyperuricemia and the associated metabolic syndrome. This study suggests novel therapeutic targets against hyperuricemia from a new perspective.

## Data Availability Statement

The RNA sequencing data have been deposited in GEO public repositories accession GSE143342. 16s RNA date was deposited in BioProject, ID: PRJNA600173.

## Ethics Statement

The studies involving human participants were reviewed and approved by the ethical committee of the Affiliated Hospital of Qingdao University. The patients/participants provided their written informed consent to participate in this study. The animal study was reviewed and approved by Animal Research Ethics Committee of the Affiliated Hospital of Qingdao University.

## Author Contributions

SX designed the study and revised the manuscript. QL and DX performed the experiments and wrote the manuscript. XY contributed significantly to the analysis. XL, WY, and PZ helped with critical discussions. XZ and GY revised the manuscript. All the authors contributed to the article and approved the submitted version.

## Conflict of Interest

The authors declare that the research was conducted in the absence of any commercial or financial relationships that could be construed as a potential conflict of interest.

## Footnotes

1 http://www.R-project.org/

2 http://www.ccb.jhu.edu/software/hisat

3 http://bowtie-bio.sourceforge.net/Bowtie2/index.shtml

4 http://deweylab.biostat.wisc.edu/RSEM
